# Comparison of cortical versus cancellous bone fixation in tendon-to-bone healing with a rat trans-calcaneal suture model for Achilles tendon sleeve avulsion

**DOI:** 10.1186/s13018-022-03469-8

**Published:** 2023-01-05

**Authors:** Shang Gao, Chao Hu, Yunjiao Wang, Jiqiang Zhang, Kanglai Tang

**Affiliations:** 1 Department of Orthopaedics/Sports Medicine Center, State Key Laboratory of Trauma, Burn and Combined Injury, Southwest Hospital, Third Military Medical University, Gaotanyan Street. 30, Shapingba District, Chongqing, 400038 China; 2 Department of Neurology, Third Military Medical University, Chongqing, China

**Keywords:** Achilles tendon sleeve avulsion, Tendon-to-bone healing, Trans-calcaneal suture technique, Rat model, Cancellous bone, Macrophage

## Abstract

**Background:**

Trans-calcaneal suture technique is an economical and effective method for repairing Achilles tendon sleeve avulsion. Whether cancellous bone fixation upon this technique could accelerate tendon-to-bone healing is unknown. The purpose of this study is to compare the effect of cortical versus cancellous bone fixation on tendon–bone healing with a novel rat trans-calcaneal suture model.

**Methods:**

Trans-calcaneal suture treatment was carried out on the right hindlimb in male Sprague–Dawley rats (*N* = 80). They were randomly divided into the cortical group (Achilles fixed to the calcaneal cortical bone, *n* = 40) and the cancellous group (Achilles fixed to the calcaneal cancellous bone, *n* = 40). Gait analysis and immunohistochemistry were performed 1, 4, 7, and 14 days after the operation. Gross observation, biomechanical analysis, micro-CT, and histological analysis were performed 4 and 8 weeks after surgery. Independent-samples *t* tests were used for comparison between groups.

**Results:**

At 1, 4, and 7 days, the swing time of the affected limb in the cancellous group decreased, while the duty cycle, the maximum contact area, the print area, and the mean intensity increased significantly. The cross-sectional area of the tendon–bone junction in the cancellous group was smaller, and the failure load and stiffness were higher 4 weeks after the operation. The cancellous group showed more proportion of new bone and a relatively well-organized and dense connective tissue interface with better fibrocartilage-like tissue at 4 weeks after the operation. The ratio of ED2 + macrophages in the cancellous group was significantly higher than in the cortical group on 1, 4, 7, and 14 days. There were no significant differences in gait at 2 weeks, in appearance, biomechanics, new bone formation, and histology at 8 weeks after surgery between the two groups.

**Conclusion:**

In the new rat trans-calcaneal suture model, cancellous fixation can accelerate tendon-to-bone healing in the early stage, which perhaps is related to the abundant bone marrow tissue in the cancellous bone that modulates the inflammatory processes.

**Supplementary Information:**

The online version contains supplementary material available at 10.1186/s13018-022-03469-8.

## Background

The Achilles tendon sleeve avulsion refers to the tendon avulsed from the posterior calcaneus as a continuous sleeve, without bony avulsion or with only a small amount of bone tissue. The residual tissue on the calcaneus is insufficient for direct suture with the distal end of the Achilles tendon [1–3]. Although the probability of this injury is much less than the rupture of the midsubstance of the Achilles tendon, it is challenging for surgeons. Because there is neither enough tendon tissue left on the calcaneus for direct repair with the proximal tendon nor enough bone tissue on the avulsion tendon for bony fixation of the calcaneal tubercle. And the healing of tendons to bone is complex and carries a bad prognosis [4–6].

Bibbo was the first to use the trans-calcaneal suture technique for repairing this type of injury and was associated with good functional results [1]. This technique is more economical than anchor fixation and avoids the risk of implant-related complications [5, 7]. Although several studies have modified this surgical treatment [3, 5, 7–9], the literature is still deficient and does not provide a strong and definite treatment [5].

In the studies of more common rotator cuff tears, more and more scholars have paid great attention to the biological repair of the tendon-to-bone interface (TBI) rather than simple anchoring fixation to improve the fixation intensity [10]. The biological aspects of tendon attachment to bone surfaces have been analyzed in some studies of rotator cuff tears [11–13], but rarely in Achilles tendon sleeve avulsion. In this study, we established a new rat trans-calcaneal suture model. We used this model to compare the tendon–bone healing effects between cortical bone fixation and cancellous bone fixation. We hypothesized that the cancellous bone fixation based on the trans-calcaneal suture technique could accelerate the healing of the Achilles tendon to the calcaneal. In addition, to explore the possible mechanisms, the macrophages were detected in the interface according to the reports that inflammatory processes play an important role in the healing process of tendon–bone [14–16].

## Methods

### Animals

The study included 80 male Sprague–Dawley rats (weighted 200 to 250 g; age 6 to 8 weeks; provided by Experimental Animal Center, Army Military Medical University). The experiment protocol was approved by the Animal Research Ethics Committee of Third Military Medical University, China. All animals were treated according to institutional guidelines for laboratory animal treatment and care.

## Experiment design

All rats underwent Achilles tendon–calcaneus fixation with the trans-calcaneal suture technique in the right posterior foot [3]. They were randomly divided into the controlled cortical bone fixation group (cortical group, *n* = 40) and the cancellous bone fixation group (cancellous group, *n* = 40).

**Fig. 1:**
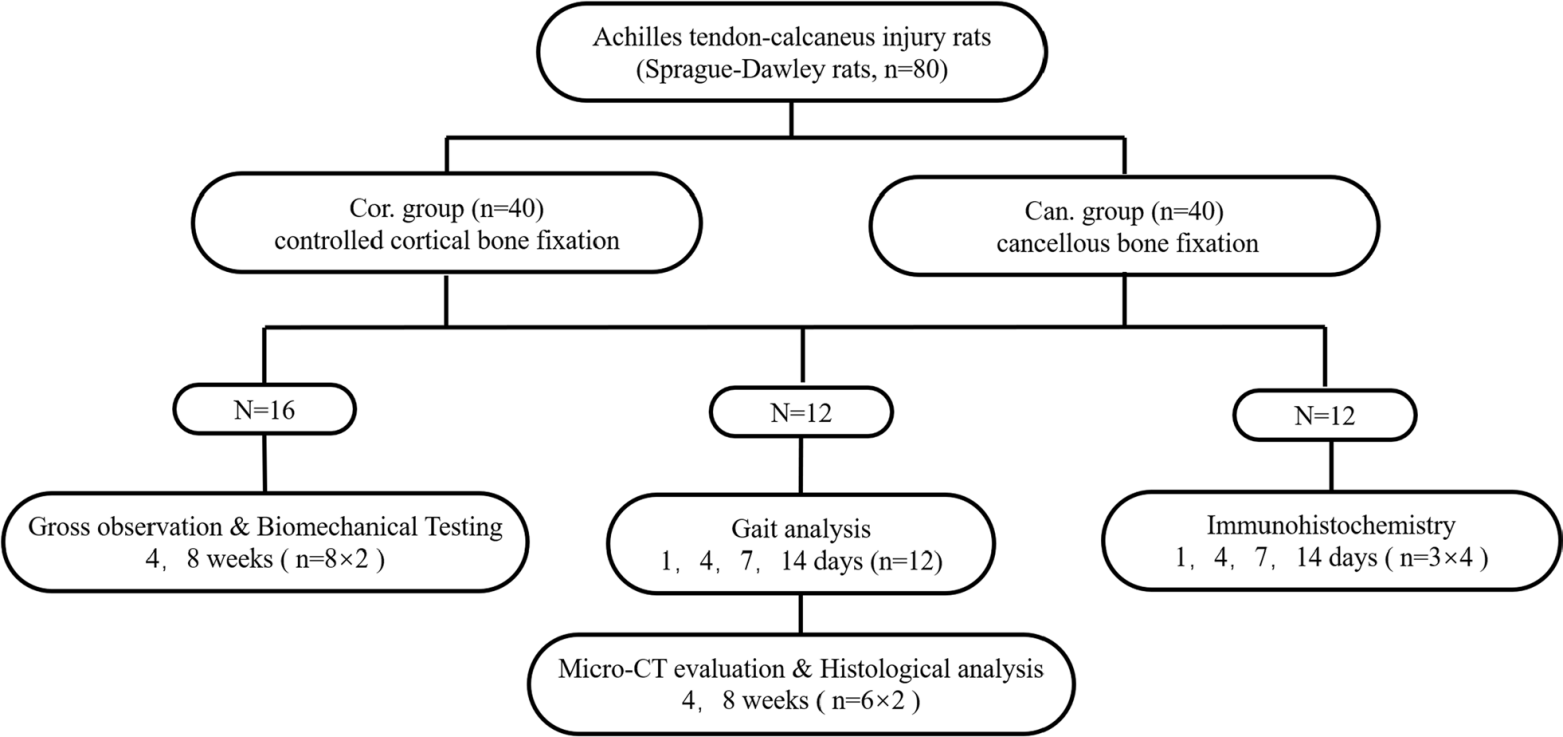
Experiment design. Cor.group: the controlled cortical group, Achilles tendon was fixed on the calcaneal cortical bone. Can.group: the cancellous group, Achilles tendon was fixed on the calcaneal cancellous bone

Sixteen rats in each group were killed at the end of 4 and 8 weeks after the operation for gross observation and biomechanical tests. Twelve rats in each group were observed by gait analysis at 1, 4, 7, and 14 days postoperatively. They were killed at the end of weeks 4 and 8 after the operation for micro-CT evaluation and histological study. Twelve rats in each group were used to detect the number of ED1-positive and ED2-positive macrophages by immunohistochemical at 1, 4, 7, and 14 days after the operation (Fig. 1).

## Surgical procedure

**Fig. 2:**
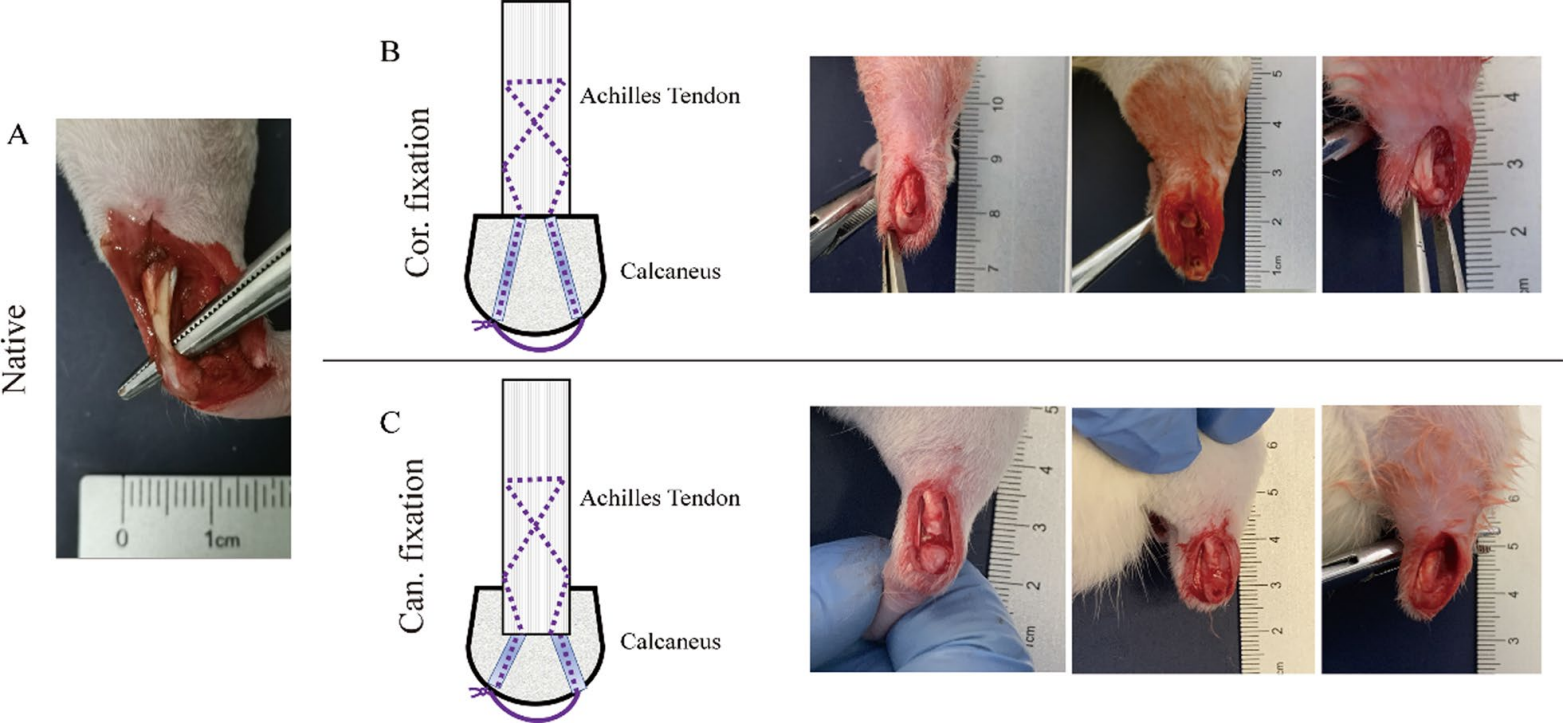
Surgical procedure of the cortical fixation and the cancellous fixation between the Achilles tendon and calcaneus. **A** The native rat Achilles tendon. **B** The Achilles tendon was fixed to the cortical bone through two drill tunnels with the suture in the cortical group. **C** The Achilles tendon was fixed to the cancellous bone within a bone hole in the cancellous group. Cor. fixation: the controlled cortical group; Can. fixation: the cancellous group

An illustration of operation protocols is briefly presented in Fig. 2. After anesthesia with isoflurane and surgical preparation, the Achilles tendon–calcaneus complex was approached through a posterior midline skin incision aseptically in the right hindlimb of each rat. The Achilles tendon–calcaneus junction was explored and incised using a sharp scalpel. The calcaneus surface and adherent fibrocartilage were partially decorticated to expose the skeletal tissues. In the cortical group, two drill tunnels were made longitudinally through the calcaneus with a 26G needle (Fig. 2B). In the cancellous group, a 1.5-mm-deep and 2-mm-diameter bone hole was made using a micro-motor drill machine (Strong 204, SAESHIN, South Korea) with a customized 2-mm-diameter drill bit in the superior posterior tubercle of the calcaneus to expose cancellous bone (Additional file 1). Then in the hole, two drill tunnels were made longitudinally through the calcaneus with a 26G needle (Fig. 2C). To avoid calcaneal fractures, the two drill tunnels through the sutures were tilted to each side of the calcaneal bottom to increase the bone bridge for the suture material and distribute the stress (Fig. 2B, C). The Achilles tendons of both groups were then sutured directly to the calcaneus via the two pre-drilled tunnels using a 4–0 absorbable suture (PGLA, Jinhuan Medical Products Co., Ltd., Shanghai, China). The suture ends were tightly tied together subcutaneously on the medial side of the calcaneal bottom, then closed the surgical incision. Rats were allowed free cage activities after the operation. The difference was that the end of the Achilles tendon in the cancellous group was inserted into the cancellous bone through a bone hole (Fig. 2 B, C).

## Gait analysis

**Fig. 3:**
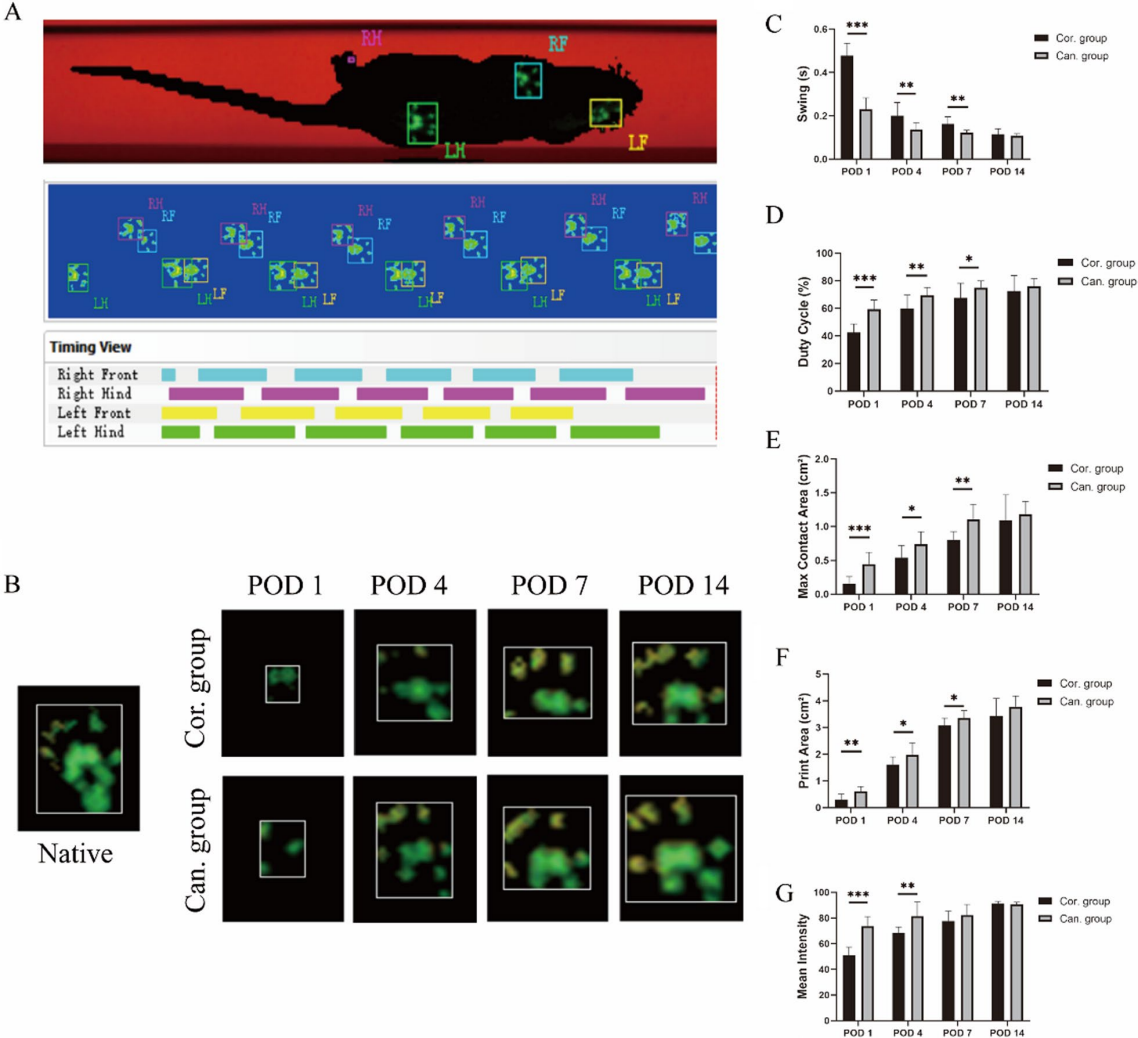
Gait analysis using the CatWalk-XT automated gait analysis system. **A** A gait cycle of a rat in the cancellous group 7 days after surgery was presented. Rat footprint marks, footprint summary, and foot touching time were shown from top to bottom. LF: left front; RF: right front; LH: left hind; RH: right hind. **B** The footprints of the right posterior foot between the two groups were shown at 1, 4, 7, and 14 days after the surgery. **C**–**G** Comparison of swing time, duty cycle, max contact area, print area, and mean intensity between the two groups at 1, 4, 7, and 14 days after the surgery. Native: Native complete footprint. Cor.group: The cortical group. Can.group: the cancellous group. POD: Postoperative day. *p < 0.05. **p < 0.01

Gait parameters were measured using the CatWalk-XT automated gait analysis system (Noldus Information Technology, Wageningen, The Netherlands). Twelve rats in each group underwent baseline testing before the operation and were tested at 1, 4, 7, and 14 days after surgery. The system consists of a runway with glass flooring and dim light illuminating the glass from the side. In a dark environment, light is reflected downward when the animal's claws touch the glass. The animals walked spontaneously along the runway toward the goal area. The camera recorded the footprint image under the sidewalk (Fig. 3A). Three consecutive trials were carried out on each animal. The images of each experiment were processed and analyzed by Catwalk-XT software on the computer to obtain average gait parameters. The most recognized and applied parameters in pain models, swing time, duty cycle, max contact area, print area, and mean intensity are used for statistics [17].

## Gross observation and biomechanical testing

Eight rats in each group were killed at weeks 4 and 8. The performance of the Achilles tendon and the peritendinous tissue were observed. The width and thickness of the Achilles tendon-to-calcaneal junction were measured with a vernier caliper. The estimated cross-sectional areas were calculated with elliptic geometry [18].

After removing the surrounding tissue of the Achilles tendon, the eight specimens in each group were fixed on a material testing device (E43.104 electronic universal testing machine, MTS, China). Both ends of the specimen were fixed with a fixture. The surface of the fixture had transverse protrusions to prevent the sample from slipping (Fig. 5A). The Achilles tendon was pulled at a 20 mm/min speed until the tendon–bone junction ruptured. Failure load (N) was recorded, and stiffness (N/mm) was calculated from the slope of the linear region of the load–elongation curve.

## Micro-CT evaluation

Six specimens in each group were fixed in 4% neutral buffered formalin and scanned by Micro-CT (Skyscan1174 X-ray Microtomography, Bruker Co., Belgium) with 12-μm voxel size at weeks 4 and 8. N-recon software was used for 3D image reconstruction, and CT-AN software was used for 3D analysis. After 3D reconstruction of the images, the newly formed bone contours on the cortical surface were manually drawn on all sections from the center of the attachment site to 2 mm proximal. In addition, the newly formed bone in the bone hole was manually drawn on all sections from the attachment site to a depth of 1.5 mm for the cancellous group’s specimens. Within these areas of interest (representing the total volume), bone volume fraction (BV/TV, bone volume/total volume), bone mineral density (BMD), trabecular number (Tb.N), trabecular thickness (Tb.Th), and trabecular separation (Tb.Sp) were calculated [19].

## Histological analysis

After micro-CT analysis, each group's six Achilles tendon–calcaneus specimens were decalcified with a 1:1 mixture of 20% sodium citrate and 50% formic acid for 5 days [20]. These tissues are then dehydrated with ethanol and embedded in paraffin. Sections were made along the longitudinal axis of the Achilles tendon at the TBI with a thickness of 5 μm. HE staining (H&E), alcian blue staining (A-B), and safranin-fixed green staining (S-G) were performed and took histologic evaluation. The images viewed under a light microscope were digitally converted, and Image J was used to outline and measure the histological healing of the TBI. H&E stained sections were used to exhibit tendon bonding to adjacent tissues of the interface. The integrated optical density of the fibrocartilage staining by A-B and S-G was measured to evaluate the fibrocartilage zone formation. Two independent observers performed the histomorphometric score using the scoring system based on tendon bonding to adjacent tissues and fibrocartilage zone formation (Table 1) [21, 22].

**Table 1:** Histomorphometric scoring criteria for the assessment of fibrocartilage zone formation at the tendon–bone interface

*Tendon bonding to adjacent tissues*	
0% of the interface	0
< 25% of the interface	1
25–49% of the interface	2
50–74% of the interface	3
75–99% of the interface	4
100% of the interface	5
Fibrocartilage zone formation	
None (0% of interface)	0
Slight (< 50% of interface)	1
Moderate (> 50% of the interface, average thickness < 100 μm)	2
Substantial (> 50% of the interface, average thickness 100–200 μm)	3
Massive (> 50% of the interface, average thickness 200–500 μm)	4
Excellent (100% of the interface, average thickness > 500 μm)	5

## Immunohistochemistry

Three rats in each group were killed by carbon dioxide inhalation at 1, 4, 7, and 14 days following surgery. The Achilles tendon–calcaneus specimens were prepared with the method mentioned above in histological analysis and made into sections. After dewaxing with xylene and rehydrating by ethanol, the tissue sections were treated with a mouse two-step assay kit (mouse-enhanced polymer assay system) (Zhongshan Golden Bridge Biotechnology, Beijing, China) for immunostaining. Mouse anti-rat antibodies to ED1 + and ED2 + macrophages (Bio-Rad Laboratories Inc., California, USA) were used to locate the macrophages in the healing tendon–bone interface. The numbers of ED1- or ED2-positive cells were counted in ten random high-power fields (HPF, 50 μm *50 μm at 400 × magnification) per specimen [16, 23].

## Statistical methods

IBM SPSS Statistics 26 was used for analysis. Data were expressed as mean ± SD, and independent-samples t tests were used to compare the two groups at each time point. The two groups considered a significant difference when the *p value* was < 0.05.

## Result

### Gait analysis

The claudication of the right hindlimb was evident in both groups within 7 days after the operation. The touchdown time of the right hindlimb in the cancellous group was higher than that in the cortical group within 7 days after the process, which was manifested by the shortening of the swing time and the increase in the duty cycle (Fig. 3C–D). The foot contact area in the cancellous group was also increased compared with that in the cortical group within 7 days after the operation, which showed that the max contact area and print area were significantly increased (Fig. 3 B, E, and F). On the 1st and 4th days after the procedure, the foot mean intensity of the cancellous group was higher than that of the cortical group, but there was no significant difference between the two groups after 7 days (Fig. 3G). At 14 days after the operation, there was no claudication in the right hindlimb of rats in the two groups. And there was no statistical difference in the above parameters between the two groups (Fig. 3C–G), and the gait was the same as that of normal rats [17].

## Gross observation and biomechanical testing

**Fig. 4:**
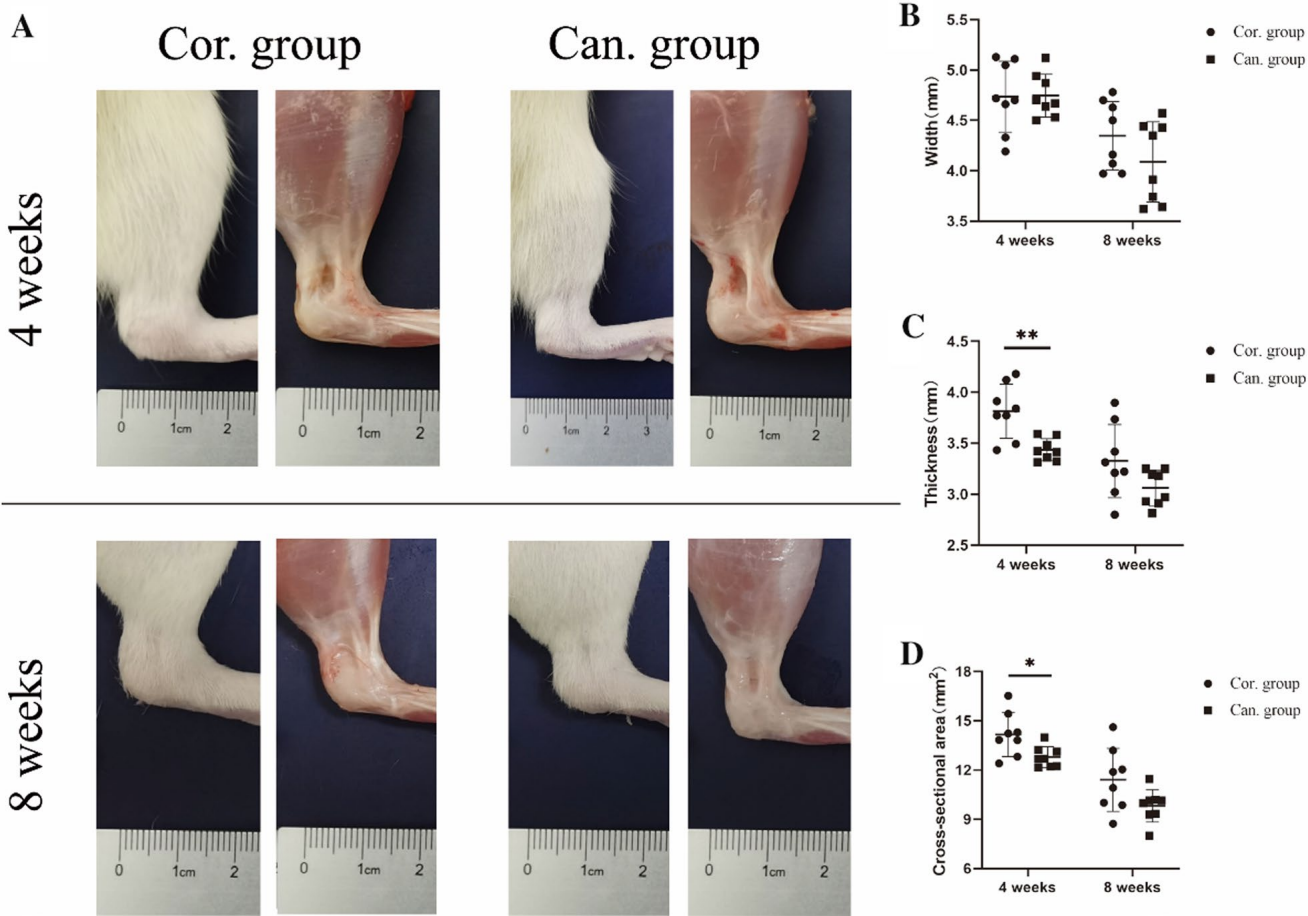
Gross observation of the healing TBI. **A** A large amount of scar tissue around the Achilles tendon and TBI in the cortical and cancellous groups and capillaries were visible on the surface at 4 weeks and were relatively reduced at 8 weeks. **B**–**D** Comparison of the width, thickness, and cross-sectional area at the Achilles tendon junction between the two groups at weeks 4 and 8. *p < 0.05. **p < 0.01

Four weeks after the operation, a large amount of scar tissue with capillaries visible on the surface was formed around the Achilles tendon and TBI in both the cortical group and cancellous group (Fig. 4A). There was no significant difference in the width of the Achilles tendon insertion between cortical and cancellous groups, but the thickness and cross-sectional area of Achilles tendon insertion in the cancellous group were significantly lower than those in the cortical group (*p* < 0.05). At 8 weeks, the scar tissue and capillaries on the surface of the Achilles tendon in the two groups were relatively reduced. The width, thickness, and cross-sectional area of Achilles tendon insertion were significantly reduced compared with those at 4 weeks, respectively. But there was no significant difference between the two groups at 8 weeks. Despite this, the cross-sectional area 8 weeks after surgery was still two to three times that of the normal Achilles insertion [24] (Fig. 4B–D).

**Fig. 5:**
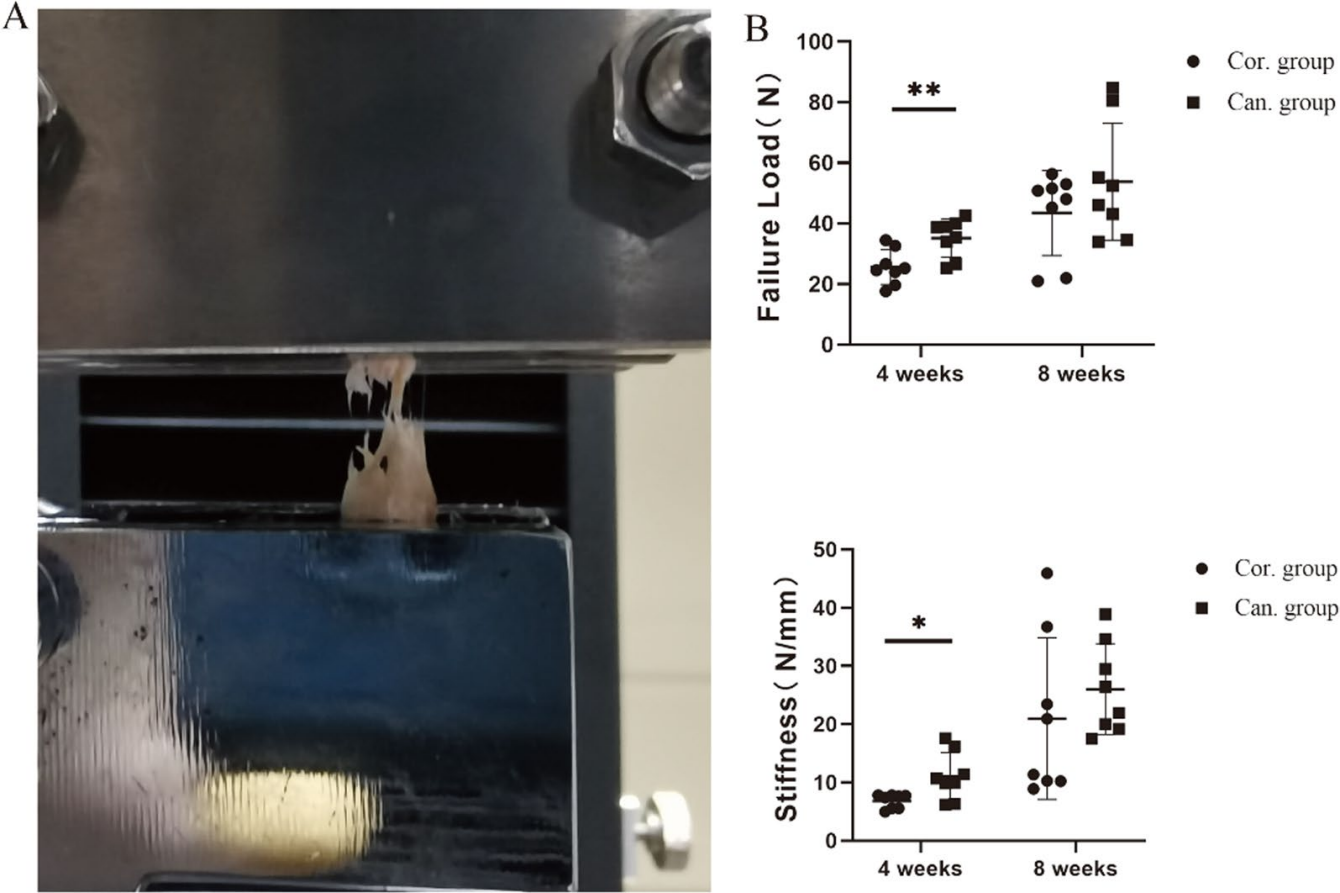
Biomechanical testing.**A** Both ends of the specimen were fixed with a fixture. The surface of the institution had transverse protrusions to prevent the sample from slipping.**B**The failure load and stiffness of the TBI in the cortical and cancellous group at 4 and 8 weeks post-operation. *p < 0.05. **p < 0.01

In the biomechanical testing, all specimens were ruptured at the tendon–bone adhesion (Fig. 5A). The failure load and stiffness of the two groups at 8 weeks were significantly more than those at 4 weeks. Four weeks after surgery, the failure load and stiffness of the cancellous group were markedly higher than the cortical group, but the difference disappeared at 8 weeks (Fig. 5B). In normal rats, the Achilles tendon is difficult to break at the attachment site, so the failure load at the BTI is greater than that in the tendon region (about 100–150 N)[24]. Therefore, it is estimated that the mean failure load of the BTI in this model at 8 weeks after the repair is not more than 40% of normal.

## Micro-CT evaluation

**Fig. 6:**
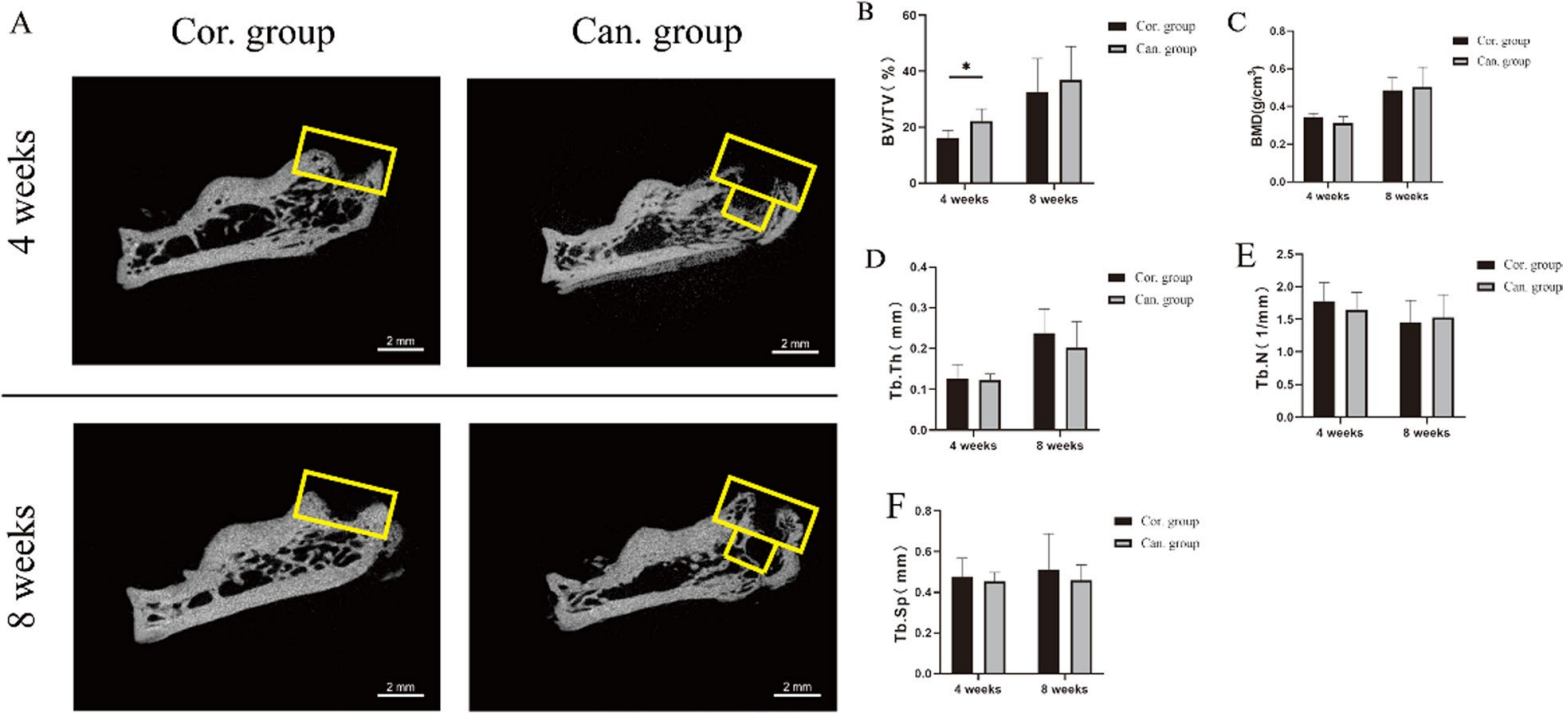
Micro-CT evaluation. **A** Sagittal micro-CT images of the calcaneus in the cortical group and the cancellous group 4 and 8 weeks after the operation. Yellow boxes represent the regions of interest. **B**–**F** Comparison of the bone volume fraction (BV/TV, bone volume/total volume), bone mineral density (BMD), trabecular number (Tb.N), trabecular thickness (Tb.Th), and trabecular separation (Tb.Sp) between the two groups at 4 and 8 weeks after the operation. *p < 0.05

Micro-CT evaluation showed that the fraction of new bone in the region of interest (BV/TV) in the cancellous group (22.29 ± 4.25%) was significantly higher than in the cortical group (16.27 ± 2.54%) only at 4 weeks (*p* = 0.017) (Fig. 6B). This difference disappeared 8 weeks after surgery. Other indexes, such as BMD, Tb.Th, Tb.N, and Tb.Sp, showed no significant difference at 4 and 8 weeks after the operation (Fig. 6C–F).

## Histological analysis

**Fig. 7:**
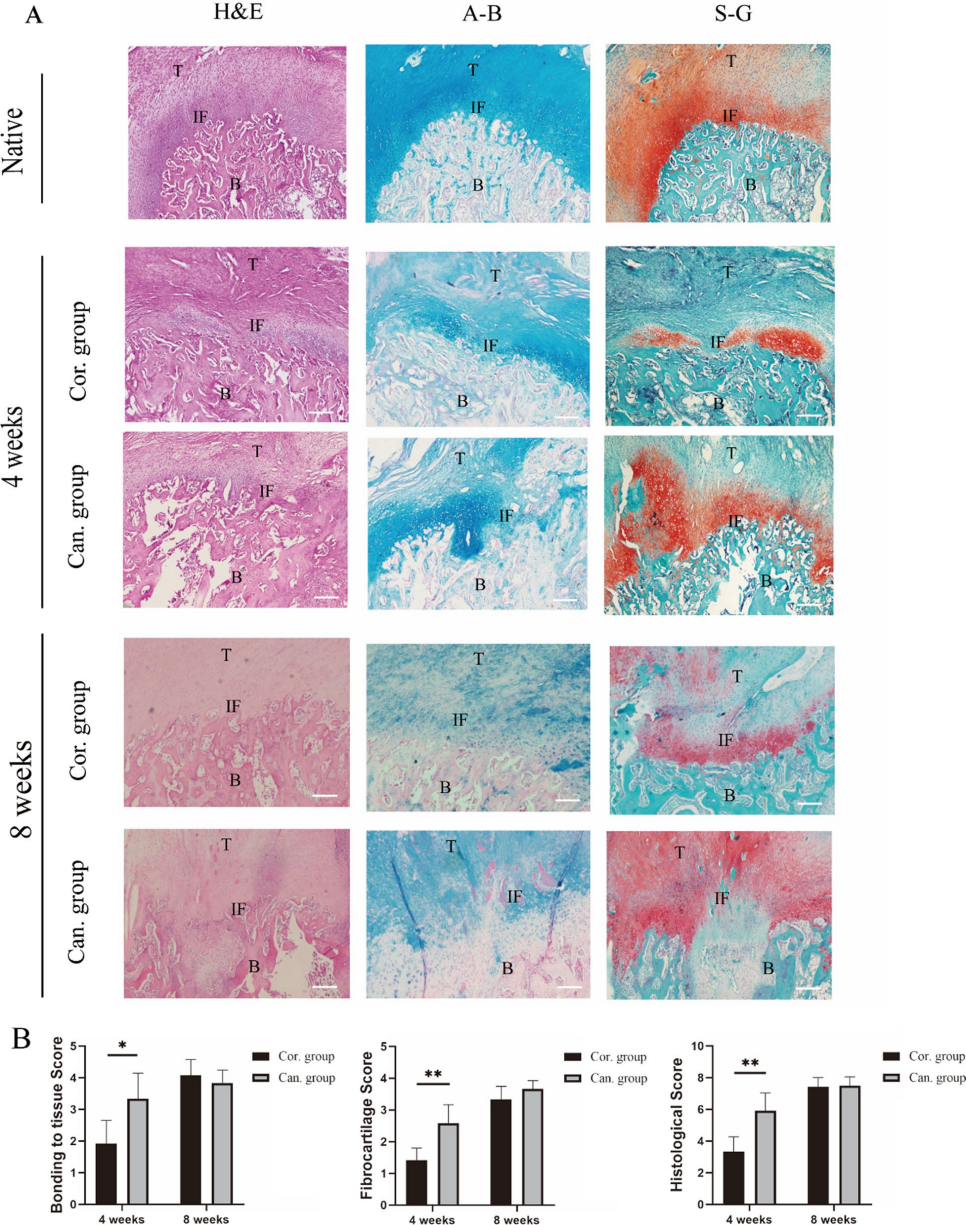
Histological analysis of the TBI. **A** Hematoxylin and eosin (H&E), alcian blue staining (A-B), and Safranin-fixed green staining (S-G) of the Achilles tendon–calcaneus healing interface of the cortical and cancellous group at 4 and 8 weeks. T = tendon, B = bone, and IF = interface. Bar = 100um. **B** Tendon bonding to adjacent tissues score, fibrocartilage zone formation score, and the histomorphometric score of the TBI in the cortical and cancellous group at 4 and 8 weeks. *p < 0.05, **p < 0.01

Histological results showed that fibrocartilage formed at the TBI in both groups at 4 weeks, and the tendons were connected to the bone tissue by Sharpey’s fibers (Fig. 7A). Compared with the cortical group, the tendon in the cancellous group had a denser and more orderly association with surrounding tissue, and more fibrocartilage tissue was formed. The tendon binding score (3.33 ± 0.82), fibrocartilage score (2.58 ± 0.58), and total tissue morphology score (5.92 ± 1.11) of the cancellous group were significantly higher than those of the cortical group (1.92 ± 0.74, *p* = 0.010; 1.42 ± 0.38, *p* = 0.002; 3.33 ± 0.93, *p* = 0.001).

At 8 weeks, H&E staining showed that the TBI was more well-organized and denser in both groups than at 4 weeks. A-b and S-G staining showed that the extent of the formed fibrocartilage band increased compared with that at 4 weeks in each group. However, the two groups had no significant statistical difference in each score (Fig. 7B).

## Immunohistochemistry

**Fig. 8:**
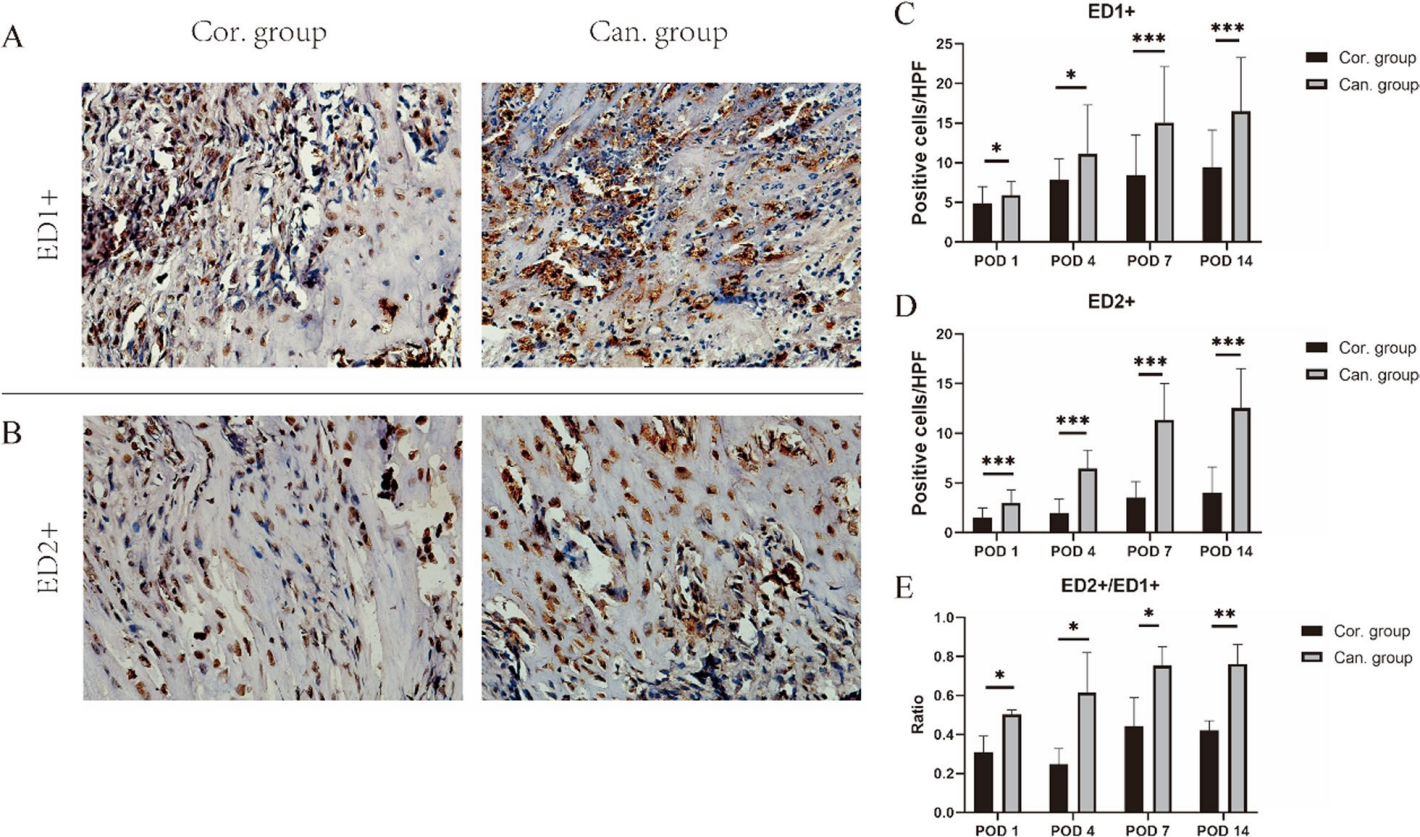
Immunohistochemistry showing ED1 + and ED2 + macrophages at the BTI. **A-B** Immunohistochemical images for ED1 and ED2 antigens at the TBI of the cortical and cancellous group 7 days after the operation at 400 × magnification. **C-D** The numbers of ED1 + and ED2 + cells in each group at 1, 4, 7, and 14 days after surgery. **E** The ratio of ED2 + cells to ED1 + cells at 1, 4, 7, and 14 days after surgery. *p < 0.05, **p < 0.01

ED1 + and ED2 + macrophages were observed in the TBI 1 day after the operation. Their numbers in both groups increased in the following days and reached relatively stable at 7 and 14 days after the process. At 1, 4, 7, and 14 days after the operation, ED1 + macrophages in the TBI of the cancellous group were significantly more than that in the cortical Group (Fig. 8A, C). The trend of the number of ED2 + macrophages was similar to that of ED1 + . At 1, 4, 7, and 14 days after the operation, the number of ED2 + macrophages at the TBI in the cancellous group was significantly higher than that in the cortical group (*p* < 0.05) (Fig. 8B, D). The ratio of ED2 + cells to ED1 + cells in the cancellous group was significantly higher than that in the cortical group at all time points (Fig. 8E).

## Discussion

Achilles tendon sleeve avulsion is much rarer than the common Achilles tendon rupture, and the related studies are limited [7]. It has been reported that the incidence of Achilles tendon sleeve avulsion accounts for 2.6–7.6% of all surgical-managed Achilles ruptures [2, 7]. The treatment is difficult because it depends on tendon fixation at the tendon–bone interface. The healing between different tissues is often slow and difficult to restore to the original anatomical structure [4, 6]. Described repair techniques for Achilles tendon sleeve avulsion include trans-calcaneal suture fixation [1, 3, 5], anchor or screw fixation [2, 8, 25], and a combination of the two [9]. Anchor or screw fixation is the most commonly used surgical technique at present. However, using implants will increase medical costs and the risk of implant-related complications. It is reported that trans-calcaneal suture fixation alone without anchors can achieve a good repair effect [3, 5]. But, clinical and basic research are limited because of the low incidence of the injury [2].

The animal model can simulate clinical injury, and on this basis, innovative treatment can be transformed into clinical practice [26]. There are few reports on animal models of Achilles tendon sleeve avulsion. Sprague–Dawley rat is a standard animal model to study tendon–bone healing, which is cheap and easy to operate. It has been widely used in rotator cuff injury repair, knee cruciate ligament reconstruction, and other fields [27–29]. We successfully established a trans-calcaneal suture fixation model of Achilles tendon rupture in SD rats and compared the difference between calcaneal cortical bone fixation and cancellous bone fixation in the healing process.

The gait analysis showed that the swing time of the operative limb was significantly decreased in the cancellous group within 1 week after the operation, and the duty cycle, the maximum contact area, and the print area were significantly higher than those in the cortical group. The mean intensity of the limb was also considerably increased in the first 4 days. It suggested that cancellous bone fixation has a relatively low pain response within 7 days after the operation. After 14 days, the two fixation methods returned to the usual walking gait, and there was no difference between the two groups. Furthermore, the gross observation and biomechanics 4 weeks after the operation showed that the cross-sectional area of the tendon and para-tendinous tissue at the insertion point was smaller and the failure load and stiffness were more prominent in the cancellous group than in the cortical group. There was no significant difference between the two groups after 8 weeks. It indicated that the cancellous group had faster-shaping speed and biomechanical recovery ability than the cortical group, but the advantage was no longer evident at 8 weeks. This is similar to the findings of St et al. [11]. They evaluated histologic and biomechanical outcomes of tendon-to-bone healing after reinserting the infraspinatus tendon onto a cortical bone or into a cancellous trough in goats and found no significant difference between groups at 6 and 16 weeks. We also found that the appearance and strength of the repaired tendon were significantly inferior to that of the normal Achilles tendon after 8 weeks, indicating that the Achilles tendon–calcaneal junction may still need a long remodeling process.

New bone and cartilage-like tissue are the main components connecting tendon and bone, which play an important role in tendon–bone healing [27]. The results showed that the proportion of new bone in the cancellous group was greater than that in the cortical group at 4 weeks. Histologically, we observed that at 4 weeks after the operation, both the adhesion of tendons to the surrounding tissue and the formation of cartilage-like tissue in the cancellous group were better than those in the cortical group, suggesting that cancellous bone fixation may promote tendon healing by promoting the formation of cartilage-like tissue. Same as the phenotype, the two fixation methods tended to be similar 8 weeks after the operation. Similar results were reported by Aoki et al. [12]. They evaluated the fibrous connections on the tendon-to-bone insertion after rotator cuff repair in the canine infraspinatus and reported that a secure fibrous connection was observed in the cancellous surface group.

Macrophages may play a key role in initiating and regulating tendon–bone healing [23]. ED1 + macrophages are related to acute inflammation. Conversely, ED2 + macrophages play a role in synthesis and metabolism in tendon repair [23, 30]. Some methods after the operation can reduce the number of ED1 + macrophages and increase the number of ED2 + macrophages and then promote the tendon–bone interface’s healing [14, 30]. In this study, it was found that after cancellous fixation of the Achilles tendon, ED1 + and ED2 + macrophages in the TBI were significantly more than those in the cortical bone fixation group. The proportion of ED2 + macrophages was increased considerably. This may be related to the abundance of fresh bone marrow in the cancellous bone. Nakagawa et al. [13] demonstrated that exposure of cancellous bone on the bone surface could increase the number of bone marrow-derived cells at the rotator cuff tendon–bone repair interface. Most of these cells came from bone marrow tissue infiltration rather than from peripheral circulation. Lu et al. [14] suggested that fresh bone marrow contains multiple progenitor cells and growth factors, such as MSCs, hematopoietic stem cells, endothelial progenitor cells, bone morphogenetic proteins, platelet-derived growth factor, transforming growth factor *β*, vascular endothelial growth factor, interleukin 8, and interleukin 1 receptor antagonist, which significantly inhibit inflammation and thus promote tendon–bone interface healing. By injecting fresh autologous bone marrow into the tendon–bone union, they increased the number of ED2 + macrophages, reduced the number of ED1 + macrophages, and significantly promoted the healing of tendon grafts and bone. Li et al. [20] found that patellar tendon fixation in the bone trough increased the contact area with the bone marrow cavity, which was conducive to cell infiltration, and the healing effect of the tendon–bone interface was better than that of bone surface tendon fixation. Besides, our results found that the promoting effect of bone marrow may be weakened with the formation of the tendon interface. Finally, the two fixation methods tend to have similar biological properties.

This study has limitations. First, we only compared cortical bone and cancellous bone fixation, not referring to different layers of the bone surface, bone marrow stimulation therapy, and so on. This is firstly limited by the difficulty of precise operation in small experimental animals, and then, exposing the cancellous bone of the calcaneus is easier than other methods in clinical practice, which has also been applied in some surgery. Second, we did not compare remodeling periods. This is because we believe that as the TBI is repairing and sealing, the bone marrow’s role in the healing process decreases. Therefore, cancellous fixation can play a positive role in early repair. What’s more, this is an animal model study. Translation to human beings needs to be proven.

## Conclusions

In summary, we established a rat trans-calcaneal suture model. In this model, cancellous fixation can accelerate tendon-to-bone healing in the early stage, which may be related to the abundant bone marrow tissue in the cancellous bone that modulates the inflammatory processes. Achilles tendon fixed on the cancellous bone may be a good clinical treatment option for the trans-calcaneal suture technique.

## Supplementary Information

**Additional file 1:** The drilling method in detail of the bone hole made in the cancellous group.

## Data Availability

The datasets used and/or analyzed during the current study are available from the corresponding author upon reasonable request.

## Abbreviations

TBI: Tendon–bone interface
SD rats: Sprague–Dawley rats
