# Supplementary material for: Comparison of cortical versus cancellous bone fixation in tendon-to-bone healing with a rat trans-calcaneal suture model for Achilles tendon sleeve avulsion
Source: J Orthop Surg Res. 2023 Jan 5;18:15. doi: 10.1186/s13018-022-03469-8 (PMC9817399; doi:10.1186/s13018-022-03469-8)

In the cancellous group, we made a 1.5-mm-deep and 2-mm-diameter bone hole using a micro-motor drill machine (Strong 204, SAESHIN, South Korea) with a customized 2-mm-diameter drill bit in the superior posterior tubercle of the calcaneus to expose cancellous bone.

To reduce the defect error caused by directly drilling with the 2.0-mm-diameter drill bit in the small calcaneus, we first used a customized 1.2-mm-diameter drill bit at a high revolving speed to create a minor cortical bone defect. Then we used the customized 2.0-mm-diameter drill bit, marked at 1.5mm from the tip, with a low revolving speed to make a standard 1.5-mm-deep and 2-mm-diameter bone hole. (The customized drill bits are shown below.)


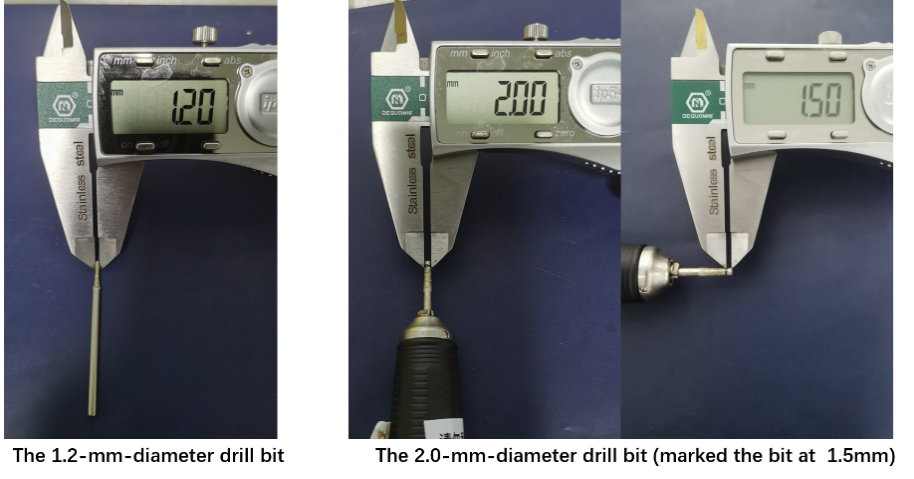

Supplement: Supplementary file 1 — Additional file 1: The drilling method in detail of the bone hole made in the cancellous group. [file 13018_2022_3469_MOESM1_ESM.docx]
